# Education and training as a key enabler of successful patient care in mass-casualty terrorist incidents

**DOI:** 10.1007/s00068-023-02232-w

**Published:** 2023-02-21

**Authors:** Patrick Hoth, Johanna Roth, Dan Bieler, Benedikt Friemert, Axel Franke, Thomas Paffrath, Markus Blätzinger, Gerhard Achatz, and the Deployment, Disaster, Tactical Surgery Section of the German Trauma Society

**Affiliations:** 1 Department of Trauma Surgery and Orthopaedics, Reconstructive and Septic Surgery, Sportstraumatology, Trauma Surgery Research Group, German Armed Forces Hospital, Oberer Eselsberg 40, 89081 Ulm, Germany; 2 Department of Radiotherapy and Radiooncology, Hospital of the State Capital Stuttgart, Kriegsbergstraße 60, 70174 Stuttgart, Germany; 3 Department of Orthopaedics and Trauma Surgery, Reconstructive Surgery, Hand Surgery, and Burn Medicine, German Armed Forces Central Hospital, Rübenacher Straße 170, 56072 Koblenz, Germany; 4 Department of Orthopedics and Trauma Surgery, Medical Faculty University Hospital Düsseldorf, Heinrich-Heine-University, Moorenstr. 5, 40225 Düsseldorf, Germany; 5 General-, Visceral-, Thoracic and Trauma Surgery, Severinsklösterchen-Hospital Köln, Jacobstr. 27-31, 50678 Cologne, Germany; 6 AUC (Academy for Trauma Surgery) of the German Trauma Society, Wilhelm-Hale-Str. 46B, 80639 Munich, Germany

**Keywords:** Education, Training, Mass-casualty incident, Mass-casualty terrorist incident, Lessons learned

## Abstract

**Background and purpose:**

The increase in terrorist attacks with sometimes devastating numbers of victims has become a reality in Europe and has led to a fundamental change in thinking and a reorientation in many fields including health policy. The purpose of this original work was to improve the preparedness of hospitals and to provide recommendations for training.

**Material and methods:**

We conducted a retrospective literature search based on the Global Terrorism Database (GTD) for the period 2000 to 2017. Using defined search strategies, we were able to identify 203 articles. We grouped relevant findings into main categories with 47 statements and recommendations on education and training.

In addition, we included data from a prospective questionnaire-based survey on this topic that we conducted at the 3rd Emergency Conference of the German Trauma Society (DGU) in 2019.

**Results:**

Our systematic review identified recurrent statements and recommendations. A key recommendation was that regular training should take place on scenarios that should be as realistic as possible and should include all hospital staff. Military expertise and competence in the management of gunshot and blast injuries should be integrated. In addition, medical leaders from German hospitals considered current surgical education and training to be insufficient for preparing junior surgeons to manage patients who have sustained severe injuries by terrorist events.

**Conclusion:**

A number of recommendations and lessons learned on education and training were repeatedly identified. They should be included in hospital preparations for mass-casualty terrorist incidents. There appear to be deficits in current surgical training which may be offset by establishing courses and exercises.

**Supplementary Information:**

The online version contains supplementary material available at 10.1007/s00068-023-02232-w.

## Introduction

Since the attacks in Madrid in 2004, Paris in 2015, and Nice in 2016, terrorist scenarios have become a relevant threat in everyday life in Europe [1].

In Germany, similar incidents such as the attack at Breitscheidplatz in Berlin in 2016, the Halle shooting in 2019 when an armed attacker attempted to enter a synagogue, and the series of attacks against Turkish businesses in Waldkraiburg in April and May 2020 have launched a public debate.

The 3rd and most recent Emergency Conference took place in Ludwigshafen on 29 November 2019. A total of 203 participants including medical and organisational leaders from local, regional and supraregional trauma centres, members of emergency medical services (EMS), and elected officials attended the conference, which focused on security in and around hospitals.

Mass-casualty terrorist incidents have special characteristics. They are fundamentally different from other mass-casualty events and are associated with severe injuries that can be considerably different from those commonly seen in everyday situations (e.g. multiple trauma after a traffic accident).

Special characteristics of mass-casualty terrorist incidents include:

- Complex and dynamic situations with high volumes of patients, for example second attacks and the uncontrolled arrival of injured and uninjured survivors at hospitals [2, 3].
- The need for cooperation and communication between hospitals and security authorities in crisis management teams and at the scene [4, 5].
- The potential threat of chemical, biological, radiological and nuclear agent use (CBRN emergencies) [6].
- The essential role of the security of hospitals, critical infrastructure, and an effective and reliable hospital emergency response plan [7, 8].
- An increased incidence of penetrating injuries, especially gunshot and blast injuries [9, 10].
- A high incidence of critical life-threatening bleeding from injuries to the extremities, junctional areas, and body cavities [11].

The need to be prepared for mass casualty (terrorist) incidents was also recognized in the new edition of the German Trauma Society’s White Paper on the Medical Care of the Severely Injured (published in 2020), and as a result further training requirements have been identified.

Against this background, we investigated the question of whether the existing international literature on past terrorist attacks provides general recommendations and lessons learned which should be considered in the organisation and content of education and training for mass-casualty terrorist incidents.

In addition, we addressed the question of the extent to which current surgical training curricula in Germany prepare junior surgeons for the special aspects and challenges associated with the management of injuries sustained by victims of terrorist attacks.

Our hypothesis is that the available literature on terrorist attacks provides good guidance on training and preparation for future events. Therefore we performed a literature review to search for quality data on preparing surgeons for mass-casualty events.

Another hypothesis is that current training concepts adequately prepare German trauma centres for terrorist attacks. We want to verify this with a current questionnaire.

## Material and methods

We conducted a comprehensive review of the literature with a focus on clinical experiences and lessons learned from the terrorist incidents listed in the GTD (Global Terrorism Database) and we conducted a survey of the audience of the 3rd Emergency Conference of the German Trauma Society (DGU) meeting to determine if the lessons learned from previous events are being taught now.

Search strategy I covered Europe, the United States and Israel, the period 1970–2017, and incidents with more than 50 casualties (fatalities and injuries). Based on these criteria, the GTD identified 174 different incidents.

As an accumulation of literature became apparent from 2001 onwards, Search strategy II was additionally designed. It covered the period 2000–2017 and incidents with 10–49 casualties (fatalities and injuries). This allowed us to identify 259 further incidents. Search strategy III was a grey literature search. Grey literature is research published outside of commercial or academic publishing.

We used GTD results, i.e. the year and location of the attacks, to search the PubMed Medline and EMBASE databases.

**Table:** 

Name of city from GTD	AND	terror*	AND	(year of incident from GTD OR management OR hospital* OR lesson* OR attack* OR administration OR preparation* OR response* OR medicine OR report* OR review* OR clinical OR disaster*)

Data from the literature and the GTD were collected and processed using a spreadsheet programme (Microsoft Excel, Version 16.58, 2022, Microsoft Corporation, Redmond, United States). Frequencies were used for descriptive statistics. A pivot table was used for Excel (Microsoft Excel, Version 16.58, 2022, Microsoft Corporation, Redmond, United States) data analysis.

To evaluate the statements and lessons learned obtained from the literature review from the past with the current situation on the topic of training at German hospitals in the trauma network of the DGU, we conducted a prospective survey.

On the occasion of the 3rd Emergency Conference of the German Trauma Society on 29 November 2019, we invited medical and organisational leaders from supraregional, regional or local trauma centres (Trauma Centers Level I, II or III) as well as members of emergency medical services (EMS) in Germany to participate in a prospective questionnaire-based survey and to express their opinion on the threat of terrorism, preparedness for possible mass-casualty terrorist incidents, and hospital security. The questionnaire comprised 28 questions. Several questions addressed the expertise of surgical personnel in the management of gunshot and blast injuries and the type and frequency of exercises at their institution.

Questionnaires from 85 staff members from German trauma centres, including many medical leaders, were analysed.

Data from the questionnaire were also collected and processed using a spreadsheet programme (Microsoft Excel, Version 16.58, 2022, Microsoft Corporation, Redmond, United States).

## Results

### Literature review

**Fig. 1:**
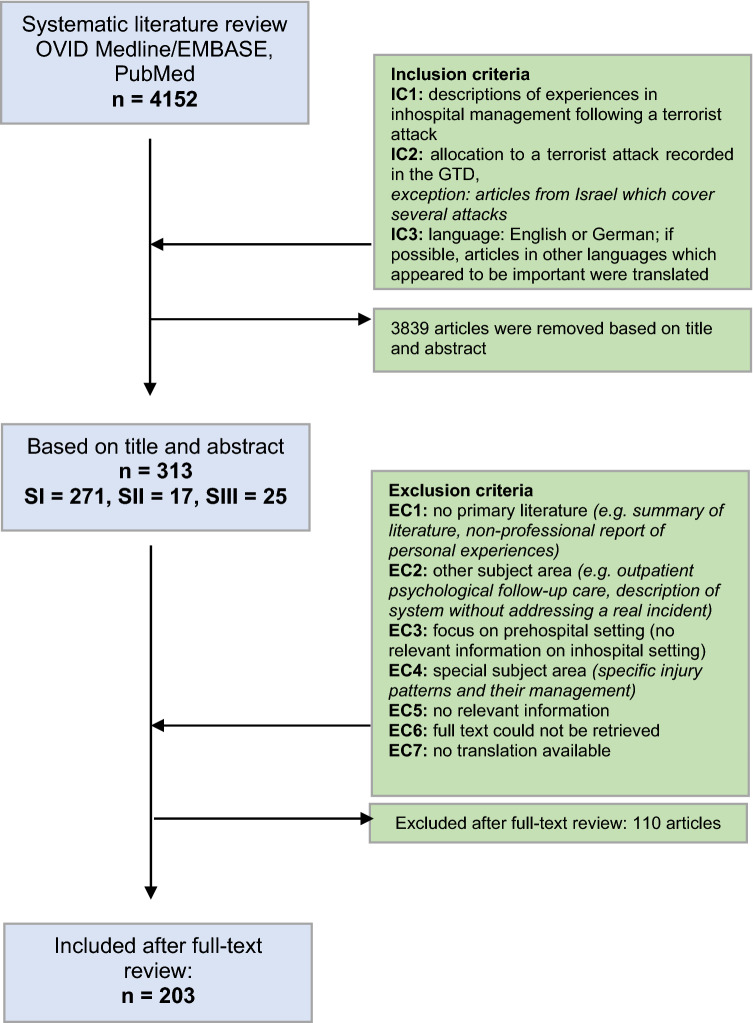
Flow chart of the search strategies including inclusion and exclusion criteria that were applied to the databases in order to identify articles appropriate for review; SI, search strategy I; SII, search strategy II; SIII, search strategy III; GTD, Global Terrorism Database; IC, inclusion criterion; EC, exclusion criterion

The search strategies identified literature on 259 attacks (6,24%). A total of 203 articles were further analysed (Fig. 1). These addressed 23 terrorist attacks recorded in the Global Terrorism Database.

Applying the inclusion criteria, 211 papers were initially identified in search strategy SI, which concerned various 21 attacks. In addition, 60 papers were found that described the situation in Israel in more detail.

With the search strategy SII, considerably more attacks were identified in the GTD. While 174 attacks were found using the SI, 259 attacks were found using the SII. Using the same search term and the same inclusion criteria, 17 additional papers were identified, distributed over six stops.

In a third search strategy, a grey literature search was carried out using the source references of all 288 papers selected for full text according to SI and SII. This made it possible to add a further 25 papers distributed over 5 of the attacks already covered by the GTD to the total number of 313.

**Fig. 2:**
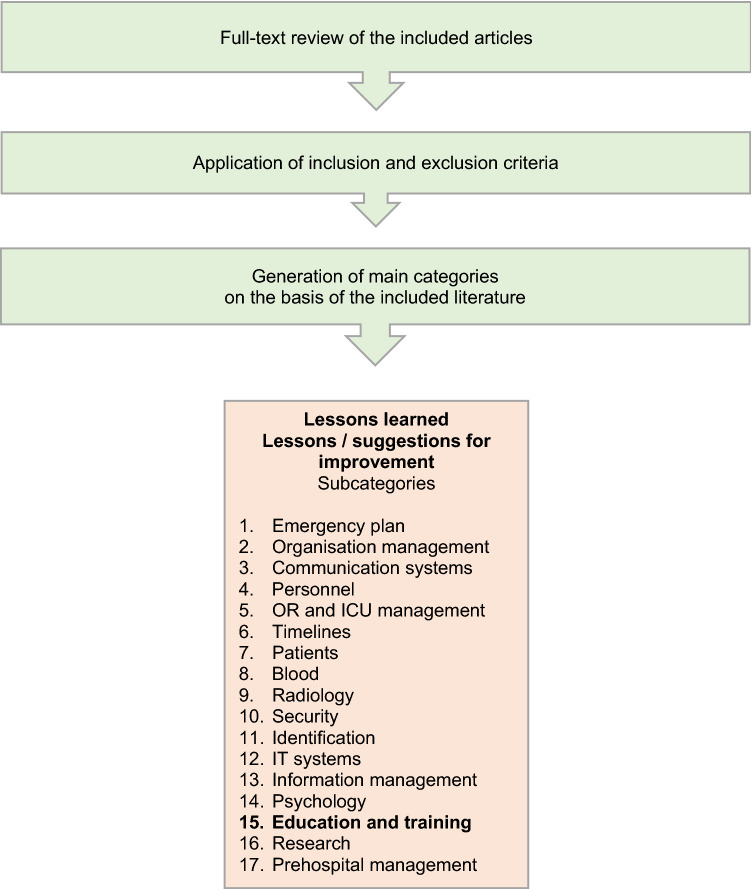
Flow chart showing the process of generating main categories for specifying the lessons learned from the literature review; OR, operating room; ICU, intensive care unit

After review, findings and statements from the articles were grouped into categories, one of which was "EDUCATION AND TRAINING" (Fig. 2). Altogether we were able to define 17 main categories.

Our next objective was to identify recurring statements and lessons learned in the literature regarding these main categories.

This approach allowed us to extract 47 statements from the literature on the main category "EDUCATION AND TRAINING" for our publication, including seven statements that were found in several articles (Table 1).

**Table 1:** Main categories for the literature search and the frequency of statements extracted from the literature and ranking based on all statements

Categories	Number of statements	Ranking (%)
Organisation management	49	13.3
Education and training	**47**	**12.7**
Personnel	27	7.3
Information management	27	7.3
Timelines	24	6.5
Identification	22	6.0
IT systems	22	6.0
Patients	21	5.7
Emergency plan	20	5.4
Psychology	18	4.9
Prehospital management	17	4.6
Communication systems	17	4.6
Security	17	4.6
Research	17	4.6
OR and ICU management	9	2.4
Blood	8	2.2
Radiology	7	1.9
All statements	**369**	**100**

OR, operating room; ICU, intensive care unit.

Table 1 shows that the main category “EDUCATION AND TRAINING” had the second most frequent mentions in our literature search.

Table 2 shows the content list of the statements on the main category “EDUCATION AND TRAINING”. We were able to sort statements with recommendations on who should provide and deliver training, who should receive the training and what the content of the training should be.

**Table 2:** List of frequent statements related to education and training on the basis of the literature review on terrorist attacks (including reference numbers)

Education and training	Number of statements	Literature
Regular training is important	14	[12–26]
Learning through simulations	1	[17]
Continuous improvement of training	1	[27]
Improvement of training through experience	1	[28]
Requesting other countries with more experience (Israel) to assist in planning for incidents	1	[29]
Hospitals that have experienced incidents have the highest level of preparedness (Oklahoma)	1	[30]
More promotion for courses	1	[31]
Who should provide education and training?		
Learning through military experience	5	[32–36]
Training at hospitals of all sizes (including small hospitals)	3	[37–39]
Military personnel should conduct courses	2	[40, 41]
Who should receive education and training?		
All personnel	3	[19, 28, 42]
Public education and information	2	[43, 44]
ICU staff	1	[13]
All surgeons and physicians	1	[45]
Radiologists	1	[46]
Non-medical personnel	1	[47]
Health care professionals	1	[43]
General surgeons	1	[31]
Content of education and training		
Paediatric patients	3	[41, 48, 49]
Use of tourniquets	2	[15, 50]
Management of typical injuries (blast injuries)	1	[45]
Injury patterns	1	[51]
Psychological trauma (including paediatric cases)	1	[52]
Administrative tasks	1	[47]
Management of large numbers of injured	1	[40, 41]
Different types of attacks	1	[40, 41]
Inhospital triage	1	[40, 41]

OR, operating room; ICU, intensive care unit.

The most frequently mentioned recommendation was that regular training is important. Another frequently recommendation was the reference to learn through military experience.

**Fig. 3:**
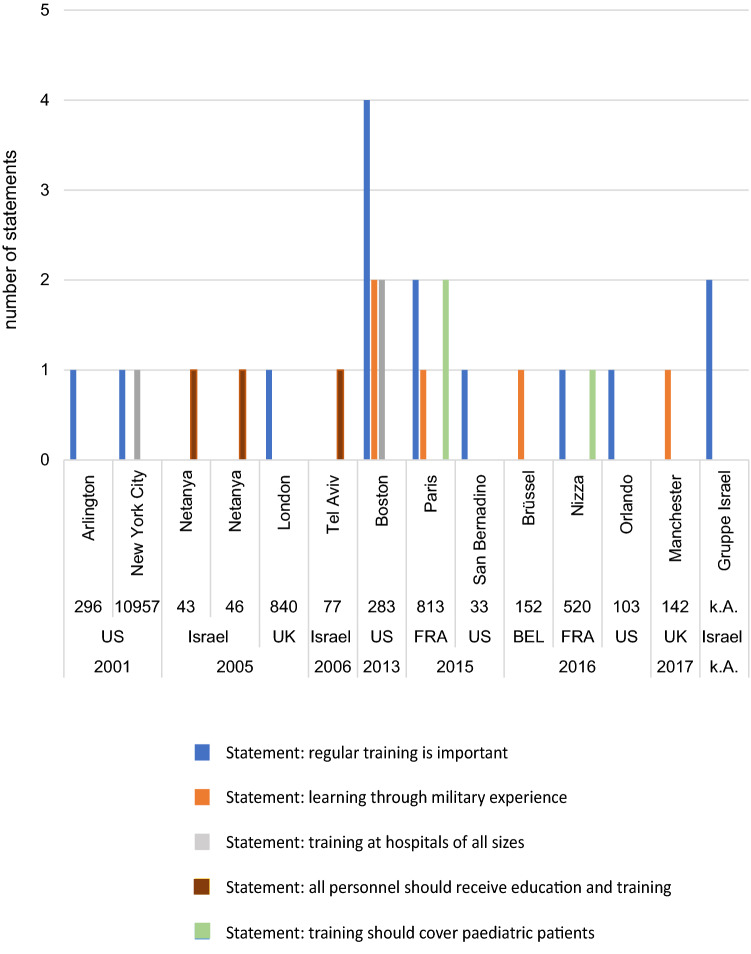
Frequency of statements related to education and training based on the literature review on terrorist attacks (including location and year of incident, number of fatalities and injuries). BEL, Belgium; FRA, France; UK,  United Kingdom; US, United States of America; k.A., no data

Figure 3 illustrates the association of identified recommendations in our main category “EDUCATION AND TRAINING” to specific attacks in the past. We have labelled the corresponding attacks with the names of the cities, the count of injured persons, the countries, the year of the attack.

Our literature review shows that especially the attacks in Boston 2013 and Paris 2015 led to several recommendations for our main category.

### SURVEY on 3rd Emergency Conference of the German Trauma Society

**Fig. 4:**
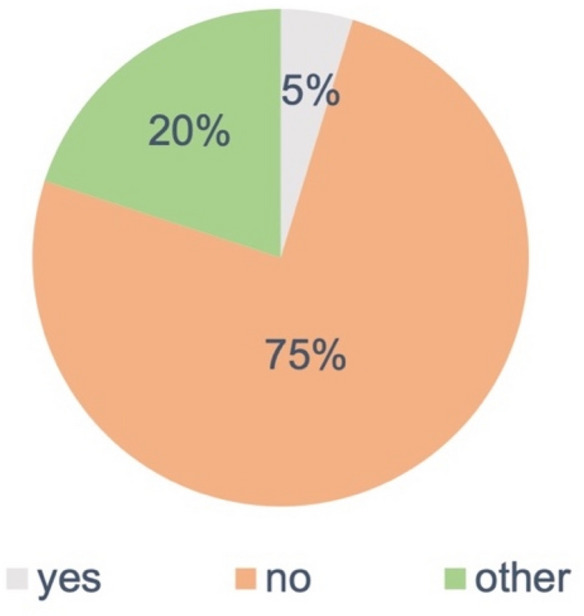
Participants’ answers to the question of whether current surgical training appropriately prepares junior surgeons for the challenges of managing injured patients after a terrorist attack

Our questionnaire included questions on whether the current surgical training in Germany adequately prepares young surgeons for the treatment of injured patients in terrorist attacks (Fig. 4). Most of respondents clearly state that this is currently not the case.

**Fig. 5:**
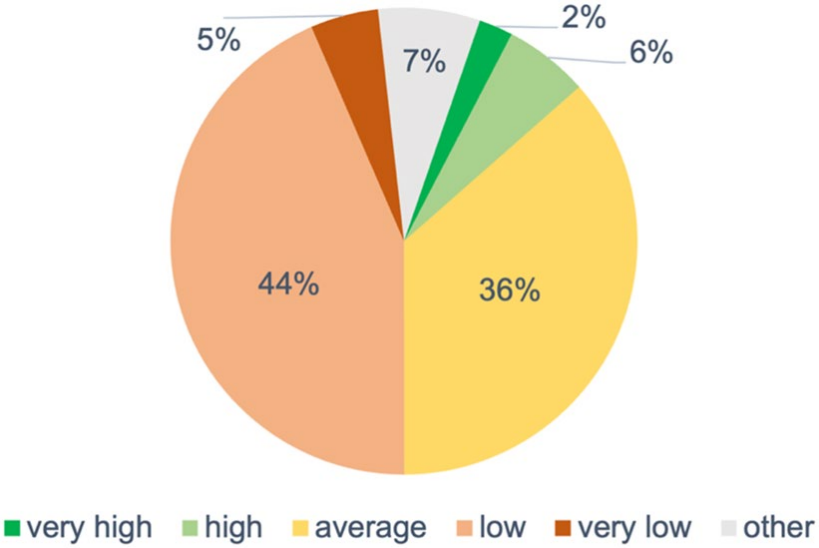
Participants’ ratings of surgical expertise in the management of terrorism-related injuries (gunshot and blast injuries) in Germany

Another question addressed the assessment of expertise of surgical colleagues in dealing gunshot and blast injuries (Fig. 5). The respondents could rate the expertise with the attributes "very high" to "very low”.

About 80% of the respondents rated the expertise either "average" or "low”.

**Fig. 6:**
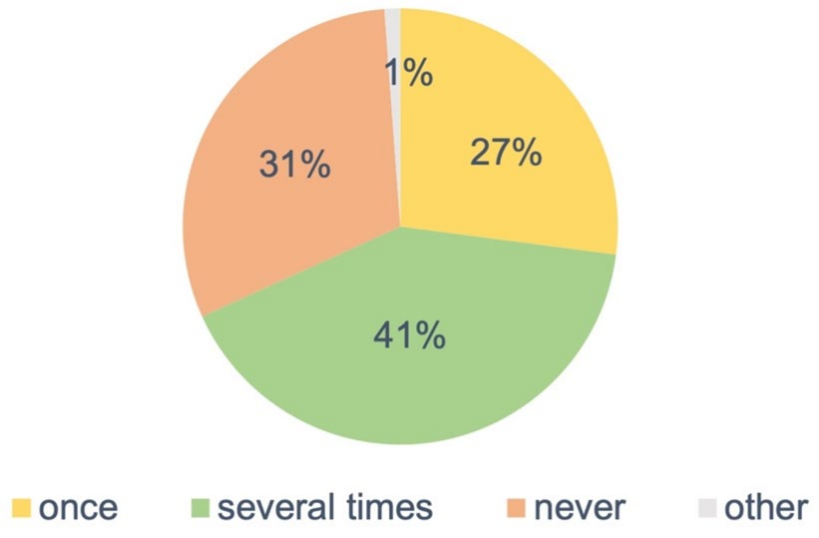
Frequency of exercises for mass-casualty incidents (MCIs) in German hospitals

In addition, we wanted to know from the participants how often mass-casualty exercises are conducted in German trauma centres (Fig. 6). More than half of the participants declared that such exercises had never been conducted before or only once.

**Fig. 7:**
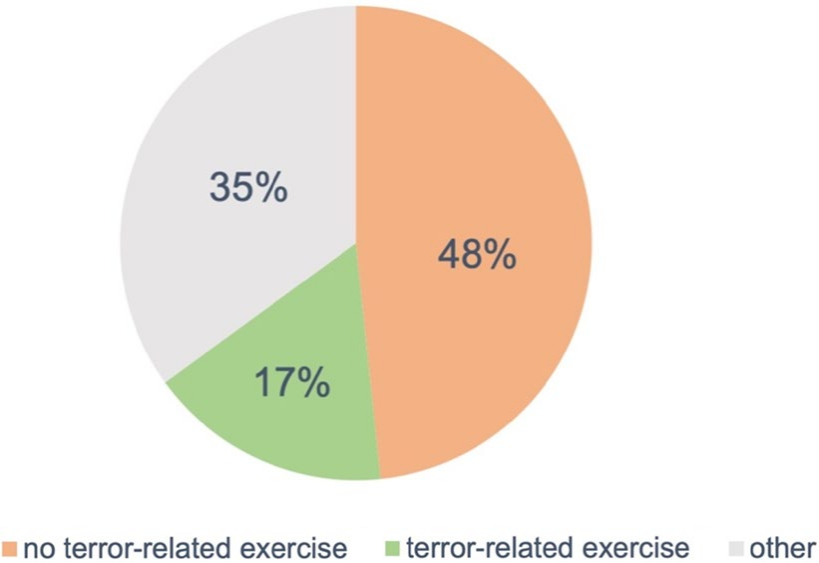
Types of mass-casualty exercises conducted at German hospitals

We were also interested in whether the conducted exercises were terror-related or not (Fig. 7). Almost half of the exercises in German trauma centres had no terror-related background. In the "other" item, all answers without specific information were collected.

## Discussion

The objective of this study was to identify possible lessons that could be learned from international experience with terrorist attacks and could be applied to improve education and training. These lessons can then be used to better prepare medical and organisational professionals for future mass-casualty terrorist incidents.

In order to identify lessons, we performed a comprehensive retrospective literature review using the Global Terrorism Database (GTD) and conducted a survey on the occasion of the 3rd Emergency Conference of the German Trauma Society in 2019 for evaluation of the lessons identified with the current situation in German hospitals of the trauma network of the DGU.

A limitation of our literature review is that only literature on 6.24% of terrorist attacks could be used for recommendations, as data for other terrorist attacks was too heterogenous or not suitable for deriving general recommendations on topics like education or training for mass-casualy incidents.

Our review of the literature shows that especially the Boston Marathon bombings in 2013 and the attacks in Paris in 2015 and Nice in 2016 led to several recommendations on how to improve education and training for mass-casualty terrorist incidents.

The subject area “EDUCATION AND TRAINING” accounted for 12.7% (47) of the 369 statements that we extracted from the terrorism-related articles that were included in our review. It was thus the second most frequently addressed subject area. This finding emphasises the importance that the international literature attaches to education and training.

The most frequent statement was that training should be provided on a regular basis. The existing literature on the Paris attacks of 2015 in particular shows that regular emergency planning, response, and resilience (EPRR) experiences have improved patient management and survival rates [1, 12]. The literature on the Boston Marathon bombings in April 2013 also underlines that regular training drills have improved patient care [16, 18, 42].

The results of the survey that we conducted at the 3rd Emergency Conference of the German Trauma Society reveal that many German hospitals that are part of the German trauma network have addressed the issue of mass-casualty exercises. Almost one third of the hospitals, however, have not yet conducted such an exercise. In addition, terrorism-related exercises accounted for less than 50% of these training events. Terrorism-related incidents, however, require special attention since they are associated with a dynamic influx of patients, specific injury patterns, and the risk of a secondary attack [9, 53]. For this reason, security in and around hospitals is a particularly important aspect of terrorist scenarios, which require effective and reliable hospital security plans [54]. Exercises that are conducted at hospitals should involve all personnel in order to address not only medical and surgical capabilities but also inhospital communication and the establishment of command structures [28, 42]. These are clear recommendations that can be derived from comprehensive analyses of the 9/11 attacks and the Boston Marathon bombings. Regardless of their size and bed capacities, all hospitals should take part in exercises since the resources of all facilities within a network will likely be rapidly overwhelmed in the event of a mass-casualty terrorist incident [37, 38]. The problem of overwhelmed resources results not only from the presence of severely injured patients who are brought in by emergency medical services but also from an uncontrolled influx of patients with minor injuries who self-refer to hospitals [55].

In addition, hospitals should prepare themselves for receiving and treating not only adults but also paediatric patients. Whenever possible, severely injured children should receive treatment and care in specialised paediatric centres. Depending on the number of injured children, however, normal trauma centres too must be prepared to provide initial care to paediatric patients [48]. From the surgical perspective, this requirement should be addressed by simulation-based training that covers the entire spectrum of treatments (DCS) for severely injured paediatric patients [49].

It was interesting to note that survey participants repeatedly emphasised that learning through military expertise can improve training and preparedness and that courses (such as simulation training) should be enhanced by experiences from the military. This is supported by international course formats such as the Medical Response to Major Incidents (MRMI®) course and the Terror and Disaster Surgical Care (TDSC®) course. Both types of courses are conducted with a significant support by military experienced personnel with deployment experience and include simulation training that helps participants improve their knowledge and skills in providing patient care in mass-casualty terrorist incidents [56, 57]. The focus of these courses is on decision-making training that enables participants to provide care to severely injured patients using limited resources and treatment strategies that are tailored to the setting, e.g. damage control surgery (DCS) and tactical abbreviated surgical care (TASC).

Triage is another extremely important aspect of training for mass-casualty (terrorist) incidents since large numbers of patients with injuries of varying severity require a rapid and accurate assessment of injuries and of the need for surgical treatment [48]. It is also essential to reliably identify patients with life-threatening injuries and to avoid overtriage and undertriage [55].

Issues such as triage in mass-casualty (terrorist) incidents are currently addressed in courses such as the aforementioned TDSC® course, which, for example, teaches the Berlin mass-casualty hospital triage algorithm [58]. A variety of prehospital and inhospital triage algorithms are available but uniform standards have not been established. The literature shows, however, that common triage algorithms are effective after a short learning phase [59].

Triages should be conducted by a senior triage coordinator outside the hospital for safety reasons. An important aspect in this context is to reduce the potential risk of second-hit attacks on medical staff [60].

By contrast, inhospital triage, i.e. categorising and prioritising patients, coordinating future patient management, and implementing the required procedures, should be performed by an emergency operational and medical coordinator (EOMC) [53].

As a result of the terrorist attacks that took place in Europe after 9/11, the use of tools such as tourniquets, which are applied in the acute treatment of wounds and injuries in combat environments, has become increasingly important in the prehospital management of severely injured civilian patients at the international level [15, 50, 61]. In our opinion, even the general public should be rigorously trained in the application of tourniquets so that they can safely use this simple measure for controlling critical extremity haemorrhage in the event of a mass-casualty (terrorist) incident.

Public education and information activities should be increased in order to prepare the public mindset for mass-casualty terrorist incidents and disaster events. Common people must learn simple procedures for responding to major incidents and must be made aware of the potential risks associated with local critical infrastructure (e.g. chemical or nuclear power plants) [32, 44].

In Germany, the Emergency and Regional Conferences of the German Trauma Society are a useful forum for increasing the awareness of issues related to mass-casualty terrorist incidents among hospitals in Germany and among the general public.

An analysis of the literature also provides possible recommendations on how to prepare for acute and long-term psychological effects experienced by health care professionals and victims who were exposed to mass-casualty terrorist incidents. Hospitals should identify appropriate response teams in advance and activate them when required in order to ensure that mental health interventions are immediately available on site in the event of an incident [52, 62]. Such teams may be composed of psychiatrists, psychologists, and nursing personnel. The composition of appropriate teams may be modified if, for example, specific care for children is required.

In addition, long-term psychological support for those affected by an incident and, if needed, for entire families should be provided. Children appear to be especially vulnerable to the effects of incidents such as those discussed here [63].

Our survey in 2019 also showed, however, that the majority of medical leaders from German hospitals considered current surgical education and training to be insufficient for preparing junior surgeons to appropriately manage patients with terrorism-related injuries [54]. For this reason, efforts are urgently needed to oblige surgeons to complete relevant courses and to take part in major/real-life exercises. This training would certify them as broadly trained trauma surgeons.

The experience and expertise of military surgeons in managing complex gunshot and blast injuries undoubtedly provide valuable and essential input for effective training that prepares participants for mass-casualty terrorist incidents [16, 35, 45]. Since these types of injuries are not commonly seen in everyday clinical routine, the acquisition of the necessary knowledge and skills through fellowships or appropriate courses can be useful. One example is the trauma fellowship programme between the German Armed Forces Joint Medical Service and the Chris Hani Baragwanath Academic Hospital in Johannesburg, South Africa. A key objective of this training programme is to provide surgical residents and specialists as well as EMS personnel the opportunity to treat an increased number of patients with gunshot and penetrating stab wounds and thus to receive training in the management of such injuries.

## Conclusions

A key lesson from major terrorist attacks in the recent past is that regular training is required and that full-scale and real-life terrorism-related exercises should be conducted and should involve all hospital personnel and structures.

The data that we obtained suggest that current initial and continuing surgical education and training does not appropriately prepare junior surgeons for the challenges of terrorism-related incidents.

Our results reflect a positive trend, namely the acceptance of the recommendation that the knowledge of military personnel should be implemented in courses and other training events that prepare hospital personnel for the special challenges of mass-casualty terrorist incidents.

Derived from the results, however, the demand clearly remains to be formulated that education and training in (German) hospitals of all sizes and medical care structures should be further improved and modified where necessary.

## Supplementary Information

Below is the link to the electronic supplementary material.

Supplementary file1 (PDF 42 kb)
