# Supplementary material for: Education and training as a key enabler of successful patient care in mass-casualty terrorist incidents
Source: Eur J Trauma Emerg Surg. 2023 Feb 21;49(2):595–605. doi: 10.1007/s00068-023-02232-w (PMC10175327; doi:10.1007/s00068-023-02232-w)
Supplement: Supplementary file 1 — Supplementary file1 (PDF 42 kb) [file 68_2023_2232_MOESM1_ESM.pdf]

1. demographic data:

gender:

☐ female ☐ male

age:

☐ 20 - 30 ☐ 30 - 40 ☐ 40 - 50 ☐ 50 - 60 ☐ > 60

2. professional position:

☐ chief physician / physician in senior position ☐ senior physician ☐ specialist ☐ assistant physician ☐ employee of a hospital administration

☐ ambulance worker ☐ nursing worker ☐ political worker ☐ other: ______________________

3. which hospital structure do you come from?

☐ supraregional trauma centre ☐ regional trauma centre ☐ local trauma centre ☐ other facility: ______________________

22. has your hospital/facility already conducted exercises on mass casualty incidents (MCI) as part of its emergency preparedness and response?

☐ yes, once ☐ yes, several times ☐ never

If yes, which ones:

☐ mass-casualty incident (MCI) ☐ terror-related MCI ☐ other

27. How do you estimate the surgical expertise for the treatment of terror-related injuries (gunshot and explosion injuries) in Germany?

☐ very high ☐ high ☐ average ☐ low ☐ very low

☐ no assessment

28: Do you think that surgical training in Germany provides the necessary contents to adequately prepare the next generation for the challenges of caring for the injured after a terrorist attack?

☐ yes ☐ no ☐ no assessment

Fig. 8:

Excerpt from the questionnaire to the participants of the 3rd Emergency Conference 2019 of the German Society for Trauma Surgery
